# Neointimal Hyperplasia and Restenosis: Epidemiology, Pathophysiology, Animal Models, Clinical Features, Diagnosis, and Management

**DOI:** 10.1002/mco2.71064

**Published:** 2026-10-19

**Authors:** Kaixu Wang, Daiyu Zhang, Yu Hu, Yixin Zhao, Xinling Wang, Long Jiang, Meixiu Jiang

**Affiliations:** ^1^ The QUEEN MARY School Jiangxi Medical College Nanchang University Nanchang Jiangxi People's Republic of China; ^2^ Jiangxi Province Key Laboratory of Bioengineering Drugs the National Engineering Research Center for Bioengineering Drugs and the Technologies Institute of Translational Medicine Jiangxi Medical College Nanchang University Nanchang Jiangxi People's Republic of China; ^3^ Division of Cardiology Department of Internal Medicine the Second Affiliated Hospital Jiangxi Medical College Nanchang University Nanchang Jiangxi People's Republic of China

**Keywords:** clinical manifestations, neointimal hyperplasia, pathological mechanisms, preclinical animal models, therapeutic interventions

## Abstract

In‐stent restenosis (ISR) remains a major complication following vascular interventions, with neointimal hyperplasia representing a central pathological process that can adversely affect postoperative outcomes. Accumulating evidence indicates that neointimal hyperplasia is driven by vascular smooth muscle cell (VSMC) phenotypic transition, endothelial dysfunction, inflammation, and multiple forms of programmed cell death. Despite substantial advances, the underlying pathogenic mechanisms remain incompletely understood. This review comprehensively examines neointimal hyperplasia from the perspectives of epidemiology, pathophysiology, preclinical animal models, clinical manifestations, diagnostic approaches, and therapeutic strategies. Particular attention is given to the emerging associations between recently characterized forms of programmed cell death, including ferroptosis and cuproptosis, and neointimal hyperplasia. In addition, commonly used preclinical animal models are reviewed, together with potential therapeutic approaches such as colchicine and vasostatin‐2 and their underlying mechanisms. The diagnostic roles of invasive imaging and noninvasive modalities in evaluating neointimal hyperplasia are discussed, with emphasis on their respective advantages and limitations. Furthermore, current stent‐based strategies, pharmacological therapies, and alternative interventions for ISR are summarized, along with critical consideration of their limitations and potential avenues for improvement. By integrating mechanistic evidence with available therapeutic options, this review aims to provide a comprehensive theoretical framework for the clinical management of ISR.

## Graphical abstract

The overall graphical diagram to summarize the pathological characteristics, pathogenesis, clinical manifestation, preclinical animal models, diagnostic methods, and intervention strategies for neointimal hyperplasia.

Neointimal hyperplasia results from excessive proliferation and intimal migration of vascular smooth muscle cells (VSMCs) in the vascular media, which ultimately induce vascular luminal stenosis. This pathological process is driven by multiple key factors, including VSMC phenotypic transition, endothelial dysfunction, and programmed cell death, further triggering adverse cardiovascular clinical events. Preclinical studies of neointimal hyperplasia mainly adopt surgical models established in C57BL/6J mice and New Zealand White rabbits, with diagnostic approaches divided into invasive and non‐invasive imaging modalities. Current therapeutic strategies cover mainstream clinical interventions and emerging promising regimens. Conventional treatments include vascular stenting and pharmacological agents to suppress VSMC proliferation and inflammatory responses via regulating related signaling pathways. Otherwise, emerging technologies such as stem cell therapy have also shown potential for treating neointimal hyperplasia.

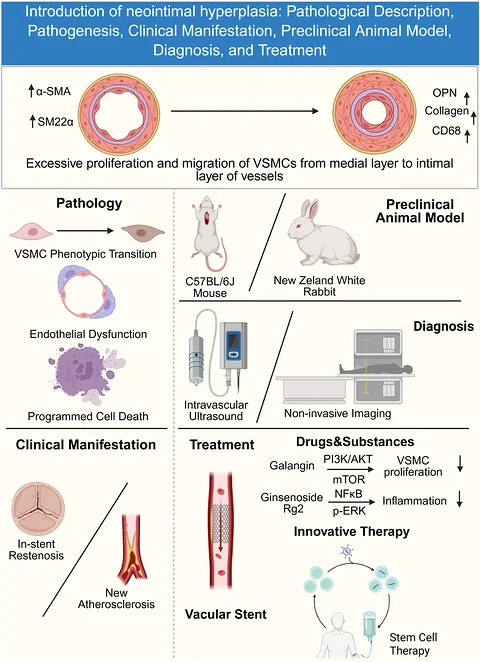

## 1 Introduction

Neointimal hyperplasia is a common pathological hallmark of restenosis following arterial injury and vascular intervention and can substantially compromise postoperative outcomes [1]. VSMCs, which reside primarily within the tunica media, play essential roles in maintaining vascular tone, elasticity, and systemic blood pressure [2]. Accumulating evidence indicates that neointimal hyperplasia is driven by a complex regulatory network centered on VSMC phenotypic transition. Extensive crosstalk among the pathological pathways involved further contributes to neointimal formation and progression.

Current clinical strategies for limiting neointimal hyperplasia primarily focus on suppressing VSMC proliferation and migration, preserving endothelial function, and modulating the local vascular immune microenvironment. Although these approaches can achieve satisfactory short‐term efficacy, long‐term outcomes remain limited by complications such as late ISR, neoatherosclerosis, and postprocedural thrombosis. These limitations underscore the need to identify additional therapeutic targets and develop more effective interventions for neointimal hyperplasia.

This review systematically summarizes the established and emerging mechanisms underlying neointimal hyperplasia and the corresponding therapeutic strategies across multiple dimensions, including epidemiology, pathophysiology, animal models, clinical manifestations, diagnostic approaches, and treatment. Particular emphasis is placed on linking pathogenic mechanisms with clinical management across the continuum from vascular injury to restenosis. Moreover, the review examines the relationship between programmed cell death and neointimal hyperplasia, with the aim of providing new perspectives for the prevention and treatment of restenosis following vascular interventions.

## 2 Epidemiology

Strictly speaking, population‐based prevalence estimates for neointimal hyperplasia in the general population are unavailable because it is a pathological response that typically develops following vascular injury. Neointimal lesions can broadly be considered in two settings: stent‐associated neointimal hyperplasia after vascular intervention and neointimal tissue formation within atherosclerotic lesions. Before the widespread use of stents, the incidence of restenosis associated with neointimal hyperplasia after plain old balloon angioplasty ranged from 32% to 55%. The introduction of bare‐metal stents (BMSs) reduced this rate to approximately 17%–41% [3], while drug‐eluting stents (DESs) further decreased the incidence to below 10% [4].

ISR, a major clinical consequence of excessive neointimal hyperplasia, has been reported in approximately 20%–27% of patients following percutaneous coronary intervention (PCI) and occurs predominantly in men older than 70 years. Comorbidities such as diabetes mellitus, dyslipidemia, and hypertension further increase the risk of ISR [5].

Race‐stratified analyses have shown that Black patients undergoing DES implantation have a significantly higher incidence of stent thrombosis than White patients (5.6% vs. 1.1%, *p* = 0.002) [6]. By contrast, subgroup analyses comparing Asian and White populations have indicated that, after adjustment for potential confounders such as diabetes, vascular anatomy, and lesion characteristics, race is not an independent predictor of ISR, with no statistically significant differences in ISR risk between these groups [7]. Overall, current evidence suggests that apparent racial or ethnic disparities in ISR are more likely attributable to differences in the distribution of baseline risk factors, such as diabetes and small‐vessel disease, than to direct effects of ethnicity on VSMC proliferation or neointimal hyperplasia.

## 3 Pathophysiology

VSMCs are the principal cellular contributors to neointimal hyperplasia. Under physiological conditions, they predominantly maintain a quiescent contractile phenotype characterized by limited proliferative and migratory activity. In response to vascular injury and other pathological stimuli, VSMCs undergo phenotypic dedifferentiation, accompanied by enhanced proliferation and migration. Increasing evidence also indicates that endothelial injury and alterations in the vascular immune microenvironment are important regulators of these cellular responses. Beyond these canonical mechanisms, emerging forms of programmed cell death have increasingly been implicated in the development of neointimal hyperplasia. This section summarizes the major pathological pathways involved in neointimal formation and their interactions, providing a mechanistic basis for the development of future therapeutic strategies.

### 3.1 Phenotypic Transition of VSMCs

The concept of VSMC phenotypic transition was first proposed by Campbell et al. in the 1970s [8, 9], and subsequent studies have established phenotypic remodeling as a central cellular mechanism underlying neointimal hyperplasia. In the quiescent state, VSMCs display a contractile phenotype characterized by the expression of contractile markers such as smooth muscle actin‐alpha (α‐SMA), myosin heavy chain 11 (MYH11), smooth muscle protein 22‐alpha (SM22α), and calponin [10, 11]. In response to stimuli including vascular injury, proinflammatory cytokines, and mechanical stress, VSMCs undergo dedifferentiation, with marked downregulation of contractile markers and acquisition of enhanced proliferative and migratory capacities. These changes promote neointimal expansion and ultimately contribute to luminal narrowing. Synthetic VSMCs represent the predominant dedifferentiated phenotype and secrete abundant extracellular matrix components, including collagen and elastin, as well as matrix metalloproteinases (MMPs), thereby facilitating VSMC migration into the subendothelial space and promoting vascular remodeling [12]. During this transition, VSMCs markedly upregulate synthetic phenotype‐associated markers such as osteopontin (OPN) [13]. Notably, VSMCs exhibit considerable phenotypic plasticity and may further differentiate into macrophage‐like, foam cell‐like, fibroblast‐like, and osteoblast‐like states, each of which exerts distinct effects on VSMC proliferation, migration, and vascular remodeling (Table 1).

**TABLE 1:** Markers and pathological conditions associated with VSMC phenotypes.

VSMC phenotype	Markers	Pathological phenomena	Reference
Contractile	α‐SMA, MYH11, SM22α, calponin	Normal vascular contraction, no specific pathology	[10, 11]
Synthetic	Elastin, collagen, MMP, OPN	Enhances VSMC proliferation/migration, basis for neointimal hyperplasia	[12, 13]
Macrophage‐like	CD68, LGALS3, CD11b, C3, CD74 (reduced contractile markers)	Key phenotype in neointimal hyperplasia, involved in inflammation and neoplastic endothelial proliferation	[14, 15, 16]
Foam cell‐like	CD68, CD36, LGALS3	Enhances phagocytosis, produces TNFα, IFNγ, IL‐1β to increase inflammatory response	[14, 17, 18]
Fibroblast‐like	COL1A1, FSP‐1, FN1	Weakens VSMC contractile function, enhances ECM synthesis, proliferation and migration capabilities	[19, 20]
Osteoblast‐like	OPN, RUNX2, ALP, osteocalcin, SOX9	Main factor in vascular calcification; BMP overexpression causes vascular remodeling via VSMC hyperproliferation	[21, 22, 23]

Previous studies have demonstrated that VSMC phenotypic transition is regulated by multiple factors, which are classified in this review into four major categories: transcription factors, epigenetic modifications, signaling pathways, and programmed cell death. These regulatory factors influence VSMC proliferation and migration through distinct mechanisms. Elucidating these mechanisms is important for identifying potential diagnostic biomarkers and developing targeted therapeutic strategies.

#### 3.1.1 Transcription Factors

Numerous transcription factors have been identified as the key regulators of VSMC phenotypic transition (Figure 1). Myocardin (MYOCD), a transcriptional coactivator of serum response factor (SRF), regulates smooth muscle‐specific gene expression by physically interacting with SRF homodimers bound to CArG box motifs [24]. Shi et al. reported that MYOCD deletion in mice reduced the expression of miR‐30a‐5p, a well‐characterized inhibitor of autophagy. The resulting enhancement of autophagic activity increased the degradation of VSMC contractile proteins and promoted phenotypic transition [25]. Transcription factor 21 (TCF21), a basic helix‐loop‐helix transcription factor encoded at the 6q23.2 locus, is another important regulator of VSMC phenotype [26]. Wirka et al. demonstrated that TCF21 is closely involved in VSMC dedifferentiation by promoting the transition of VSMCs from a contractile state toward a fibroblast‐like synthetic phenotype [27]. Kruppel‐like factor 4 (KLF4), a zinc‐finger transcription factor, has been reported to exert vasculoprotective effects by inhibiting angiotensin II‐induced VSMC phenotypic transition, potentially through mechanisms involving cellular autophagy [28]. As a redox‐sensitive transcription factor, NF‐κB is tightly regulated by intracellular redox status, with the accumulation of reactive oxygen species (ROS) serving as an important trigger of NF‐κB activation. Functionally, activated NF‐κB promotes VSMC dedifferentiation through the NF‐κB/mTOR/P70S6K signaling cascade, providing a mechanistic rationale for investigating NF‐κB inhibitors as potential therapeutic agents for neointimal hyperplasia [29]. In addition to NF‐κB, nuclear factor erythroid 2‐related factor 2 (NRF2) contributes to the maintenance of intracellular redox homeostasis and VSMC phenotypic stability [30]. He et al. found that NRF2 depletion in mice markedly promoted the transition of VSMCs toward a synthetic phenotype, whereas NRF2 overexpression preserved the contractile phenotype and attenuated neointimal thickening [31]. These findings suggest that NRF2 acts as a negative regulator of VSMC phenotypic transition. Octamer‐binding transcription factor 4 (OCT4), which is best known for its role in maintaining pluripotency and inducing pluripotent stem cells, also participates in the regulation of VSMC phenotype [32]. Shin et al. reported that smooth muscle cell‐specific OCT4 deficiency accelerated phenotypic transition and aggravated atherosclerotic lesions in mouse models, highlighting an unexpected role for this stem cell‐associated transcription factor in vascular homeostasis [33]. Runt‐related transcription factor 2 (RUNX2), previously identified as a marker of osteoblast‐like VSMCs, also functions as a transcriptional regulator that promotes VSMC dedifferentiation. Mechanistically, RUNX2 disrupts the interaction between MYOCD and SRF, thereby suppressing contractile gene expression and facilitating the transition toward a synthetic phenotype [34].

**FIGURE 1:**
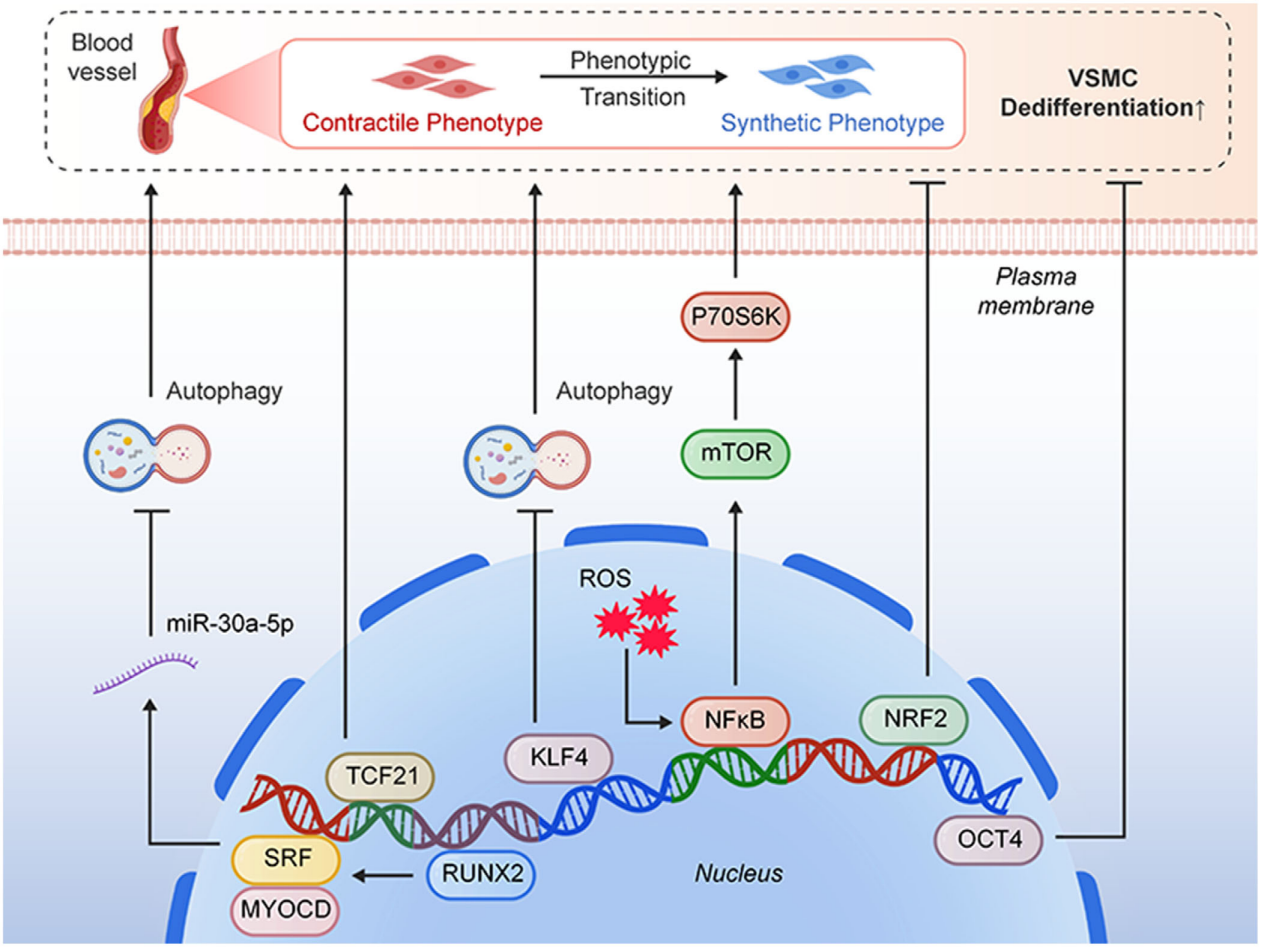
Signaling pathways regulating VSMC phenotypic transition in neointimal hyperplasia. Under pathological conditions, VSMCs primarily undergo a shift from a contractile phenotype to a synthetic phenotype, representing an enhanced process of VSMC dedifferentiation. During this process, certain transcription factors directly influence the transition. For instance, TCF21 directly promotes the shift from a contractile to a synthetic phenotype in VSMCs, while factors like NRF2 and OCT4 directly maintain the contractile state of VSMCs. Other transcription factors exert indirect effects on phenotypic transition. For instance, MYOCD and KLF4 regulate cellular autophagy to reduce degradation of contractile markers, thereby sustaining the contractile state. Conversely, NF‐κB is activated by elevated ROS levels, initiating downstream mTOR/P70S6K signaling pathways that induce VSMC transition from the contractile to the synthetic phenotype.

Collectively, these transcription factors form a complex regulatory network that bidirectionally controls VSMC phenotypic plasticity. Individual transcription factors may either stabilize the contractile phenotype or promote dedifferentiation toward synthetic, fibroblast‐like, or osteogenic states. Importantly, the activity of these transcriptional regulators is not static; their ability to bind target gene regulatory regions and reshape downstream transcriptional programs is profoundly influenced by epigenetic modifications.

#### 3.1.2 Epigenetic Modification

Epigenetic modifications are molecular changes that are heritable but reversible and do not involve changes to the DNA sequence and affect gene expression. Epigenetic changes affect chromatin structure and accessibility, and thus play an important role in the establishment of cellular identity and phenotype. In the context of phenotypic plasticity in VSMCs, the epigenetic regulation of the process may be divided into three major pathways, namely DNA methylation, post‐translational modifications of histones, and regulation via non‐coding RNA (ncRNA) molecules [35]. Among them, the epigenetic process of DNA methylation is the best studied one and has a significant role in the process of VSMC dedifferentiation. One of the enzymes responsible for the process of DNA demethylation is ten‐eleven translocation 2 (TET2), which belongs to the group of methylcytosine dioxygenases. It was shown that the knockdown of TET2 expression causes DNA hypermethylation, induction of dedifferentiation program, and development of synthetic VSMC phenotype. On the other hand, increased TET2 activity maintains hypomethylation of contractile genes' promoters; hence, the maintenance of the contractile gene's phenotype is promoted [36]. Histones are important structural constituents of chromatin. A nucleosome serves as the basic organization unit in chromatin. A nucleosome is made up of two molecules each of histones H2A, H2B, H3, and H4. The N‐terminal tails of histones get methylated, acetylated, phosphorylated, and ubiquitylated among other post translational modifications. These modifications play an important role in many biological processes [37]. The binding of the SRF/MYOCD transcription factor complex to the CArG binding elements is controlled by a variety of histone modifications in VSMCs [38]. Zhong et al. have demonstrated that increased levels of histone methyltransferase SMYD2 increase SRF transactivation capacity by increasing the level of HDAC3 that aids in the deacetylation of SRF [39]. Disruptor of telomeric silencing 1‐like (DOT1L) is a protein that acts as an epigenetic writer that carries out methylation of histone H3 at lysine 79 (H3K79). Its methyltransferase activity is markedly increased in the carotid arteries of rats following balloon injury. Notably, DOT1L silencing reduces α‐SMA expression, supporting an important role for DOT1L‐mediated epigenetic regulation in SMC phenotypic remodeling and neointimal development [40].

In addition to DNA methylation and histone modifications, regulation through ncRNA is another important research area. The types of ncRNAs that regulate VSMCs can be classified according to their size and role into microRNAs (miRNAs) and long non‐coding RNAs (lncRNAs). MiRNAs are highly conserved and short single‐stranded ncRNAs with a size of approximately 22 nucleotides. Previous studies have shown that miRNAs including miR‐24, miR‐26a, miR‐31, miR‐96, and miR‐146a play roles in phenotypic modulation of VSMC from contractile to synthetic. On the other hand, other miRNAs such as miR‐1, miR‐133, miR‐145, and miR‐15b/16 maintain the contractile state of the cells [41]. Apart from miRNAs, lncRNAs also play crucial roles in the phenotypic transition of VSMCs. A lncRNA is a noncoding RNA molecule having more than 200 nucleotides in its length and no protein‐coding capacity, and it possesses a unique pattern of conservation as compared to highly conserved protein coding mRNAs. Many mammalian lncRNAs retain syntenic conservation at their genomic loci despite limited full‐length sequence homology [42], and several lncRNAs involved in VSMC regulation exhibit species‐specific expression and function [43]. Emerging evidence indicates that lncRNAs can bidirectionally regulate VSMC phenotypic transition. Among lncRNAs that promote a proliferative phenotype, H19 is markedly upregulated in injured vessels [44]. By binding to miR‐107, H19 alleviates miR‐107‐mediated repression of insulin‐like growth factor 1 receptor (IGF1R), thereby activating the phosphoinositide 3‐kinase (PI3K)/protein kinase B (Akt) signaling pathway. This promotes the transition of VSMCs from a contractile to a proliferative phenotype and accelerates neointimal formation within the vascular lumen [45, 46, 47, 48]. Conversely, certain lncRNAs suppress pathological VSMC phenotypic transition. Liu et al. found that lncRNA CARMN was significantly downregulated in mouse abdominal aortic aneurysms (AAAs). Through its interaction with SRF, CARMN enhances the expression of VSMC contractile marker genes, including α‐SMA and SM22α, thereby helping to maintain the contractile phenotype [39, 49]. In summary, ncRNAs are important modulators of VSMC phenotypic plasticity, and targeting these molecules or their downstream effectors may offer potential strategies for limiting pathological neointimal hyperplasia.

#### 3.1.3 Signaling Pathways

Although numerous transcriptional regulators associated with neointimal hyperplasia have been extensively investigated, the signaling networks through which they regulate VSMC phenotype remain incompletely understood. These signaling pathways can be broadly classified according to whether they promote or suppress the transition of VSMCs toward a synthetic phenotype. For instance, studies of thoracic aortic dissection (TAD) involving serum‐ and glucocorticoid‐regulated kinase 1 (SGK1) have implicated aberrant SGK1 upregulation in pathological VSMC remodeling. Mechanistically, SGK1 promotes the transition of VSMCs toward a synthetic phenotype through the SGK1‐SIRT6‐MMP9 regulatory axis [50]. In contrast, GATA‐6, a transcription factor abundantly expressed in vascular tissue, suppresses neointimal hyperplasia primarily through the GATA‐6/CCN5 signaling axis [51]. Additional signaling pathways involved in VSMC phenotypic transition and their corresponding mechanisms are summarized in Table 2.

**TABLE 2:** Overview of signaling pathways associated with VSMC phenotypic transition.

Signaling pathway	Cellular function	Regulator	Reference
SGK1–SIRT6–MMP9	Regulate the aging process	MMP9	[50]
Lkb1–Ptbp1–PKM2	Promotes VSMC dedifferentiation	Lkb1	[52]
PI3K/Akt/mTOR	Promote synthetic phenotype	TRIM65	[47]
ROS/NF‐κB/mTOR/P70S6K	Initiate the dedifferentiation of VSMCs	ROS	[29]
GPR54/Gαq11‐dependent ERK1/2–STAT3	Promote VSMCs phenotype transition and aggravating atherosclerosis	Asprosin	[53]
TNFα–COX2–PGD_2_	Promote the phenotypic regulation of VSMCs	PPAR	[54]
RANKL/RANK–OPG	Mediated VSMC transdifferentiation and calcification	OPG	[55]
GATA6–CCN5	Inhibit neointimal hyperplasia of blood vessels	PDGF‐BB	[51]
NLRP3 inflammatory vesicles–NF‐κB	Regulate the phenotypic transition of VSMCs and drive vascular inflammation	Histone acetyltransferase, curcumin	[56]
Septin4–SIRT1–STAT3	Promote the proliferation, migration and phenotypic transition of VSMCs	Septin4	[57]
QKI–cZFP609–PDGFRβ	DAMPs aberrant VSMC plasticity and mitigates vascular remodeling	SIRT1	[58]
PLCγ1/Ca^2+^/MMP9	Promote vascular remodeling	Betulinaldehyde	[59]
Notch/RBP‐Jκ	Contributes to neointimal hyperplasia by promoting the migration of VSMCs into the venous anastomosis	FSP‐1	[60]
IMD_1‐53–_CRLR/RAMP3–PKA–α‐klotho	Inhibit vascular calcification and protect vascular endothelial function	cAMP	[61]
GSTA4–4‐HNE–MAPK	Promote SMC transition, proliferation and cell detoxification	4‐HNE	[62]

### 3.2 Endothelial Cell Dysfunction

Endothelial cells form the inner lining of blood vessels and secrete vasoactive mediators, including nitric oxide (NO) and PGI_2_, to maintain vascular homeostasis. Accumulating evidence indicates that these homeostatic and protective functions are disrupted by vascular injury, endothelial denudation, or persistent endothelial dysfunction. This disruption initiates a cascade of pathological events that ultimately promotes neointimal hyperplasia. Therefore, endothelial dysfunction is considered a key initiating event in ISR and vein graft disease. Multiple pathological processes contribute to endothelial dysfunction, among which endothelial‐to‐mesenchymal transition (EndoMT), oxidative stress, and dysregulated NO signaling play particularly important roles (Figure 2). Elucidating these mechanisms may facilitate the development of endothelial‐targeted therapeutic strategies for neointimal hyperplasia.

**FIGURE 2:**
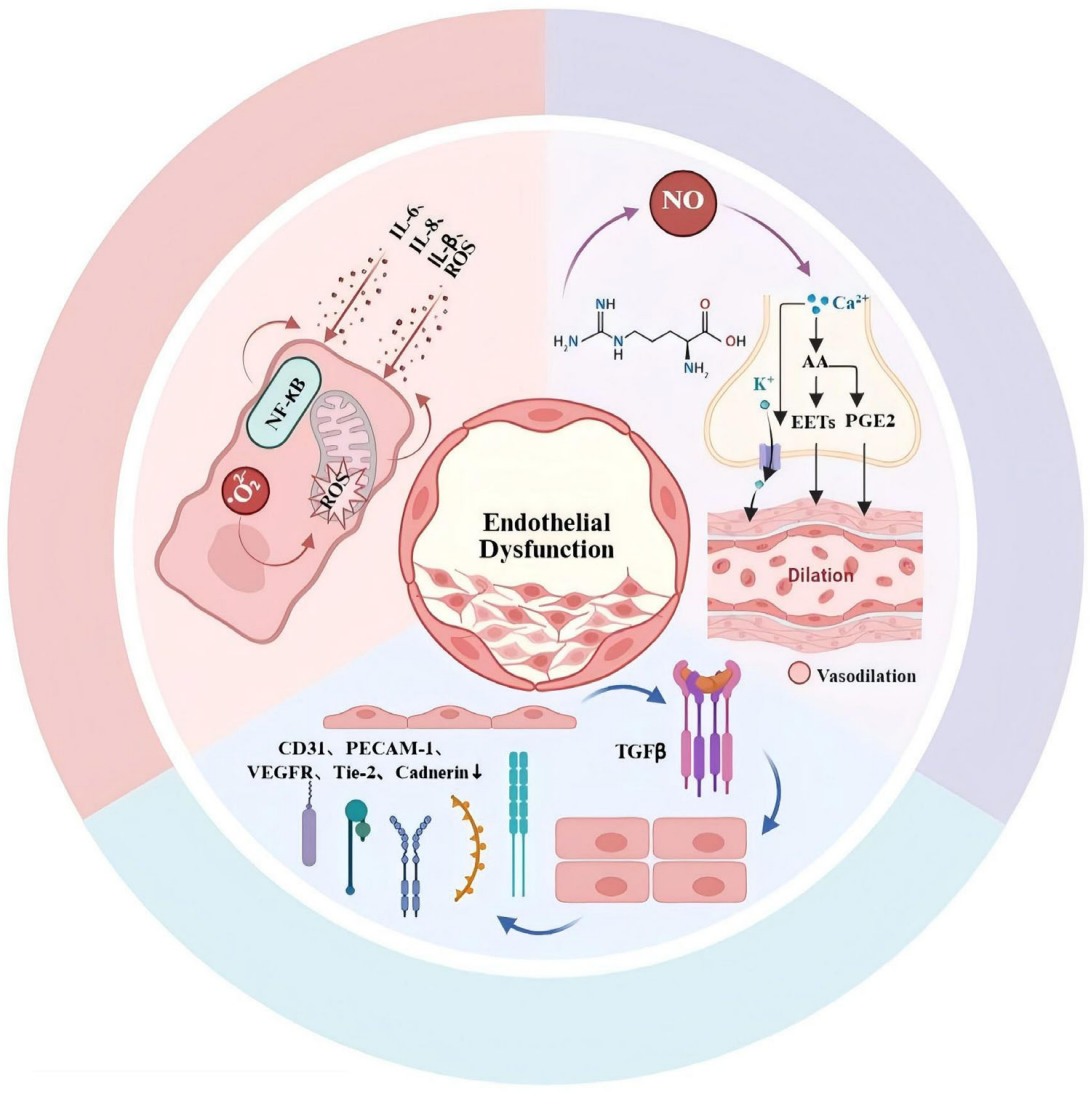
Molecular regulatory mechanisms of endothelial dysfunction. Endothelial dysfunction primarily encompasses three aspects: increased ROS production, reduced NO generation, and EndoMT. When the production of ROS increases, it will activate inflammatory factors and disrupt endothelial cells, and excessive ROS will also lead to damage of endothelial cells, ultimately leading to endothelial dysfunction. In addition, NO produced by endothelial cells exerts a vasodilatory effect by reducing intracellular Ca^2^^+^ concentrations, a process which may exert a certain protective effect on endothelial cells. Beyond these factors, enhanced EMT downregulates the expression of certain cell surface molecules, such as CD31, VEGFR, and PECAM‐1, which also impair the normal function and structure of endothelial cells, ultimately leading to dysfunction.

#### 3.2.1 Endothelial‐To‐Mesenchymal Transition

Similar to the phenotypic transition of VSMCs, endothelial cells can undergo EndoMT in response to pathological stimuli such as disturbed shear stress and stent‐induced mechanical injury, giving rise to cells with myofibroblast‐like characteristics. During this transdifferentiation process, endothelial cells progressively acquire mesenchymal properties, including enhanced migratory, proliferative, and extracellular matrix‐secretory capacities, together with increased expression of mesenchymal markers such as α‐SMA, vimentin, and fibronectin [63].

Mechanistically, accumulating evidence identifies transforming growth factor‐β (TGF‐β) as a major driver of EndoMT following vascular injury. TGF‐β binds to its receptors and induces phosphorylation of downstream Smad2 and Smad3, thereby activating transcriptional programs that promote endothelial phenotypic remodeling [64]. In addition to Smad2/3 signaling, EndoMT is regulated by transcription factors and extracellular modulators that interact with the TGF‐β pathway. For example, the transcription factor Snail1 acts in concert with the TGF‐β/Smad2 signaling axis to promote EndoMT [65], whereas the extracellular matrix protein tenascin‐X (TN‐X) reduces TGF‐β bioavailability and restrains this process [66]. Beyond the canonical TGF‐β/Smad pathway, several additional signaling cascades, including the Notch, MAPK, and Wnt pathways, have also been implicated in EndoMT [67, 68]. The major regulators of EndoMT and their mechanisms of action are summarized in Table 3.

**TABLE 3:** Signaling pathways associated with EndoMT and their specific mechanisms of action.

Pathway	Key signaling gene/protein	Specific mechanisms	Reference
TGF‐β	TGF‐β1, TGF‐βR1/2	Major EndoMT inducer and promotes post‐injury neointimal hyperplasia	[69]
	Smad2/3	Phosphorylated Smad2/3 upregulates Snail1/2 and Twist1 to drive TGF‐β‐dependent EndoMT	[70]
	Smad7	blocks Smad2/3 phosphorylation, attenuates EndoMT and limits neointima hyperplasia	[71]
NOTCH	Notch1	NICD (Notch intracellular domain) translocates to nucleus, cooperates with Smad complexes to trigger EndoMT	[72]
	Jagged2	Activated Jagged2‐Notch1 signaling elevates downstream GATA transcription factors, which synergize with TGF‐β‐Smad cascades to promote pathological EndoMT	[73]
MAPK/NF‐κB inflammatory axis	p38 MAPK, NF‐κB	Inflammation‐activated p38 and NF‐κB synergize with TGF‐β–Smad signaling, and exacerbate endothelial inflammation to amplify EndoMT	[74]
Protective KLF axis	KLF2, KLF4	Laminar shear stress‐induced KLF2/4 suppress EndoMT development, knockout of KLF2/4 will induce EndoMT	[75, 76]
SIRT1	SIRT1	Upregulates expression of KLF2 and KLF4, thus inhibiting EndoMT	[77]

#### 3.2.2 NO

NO, a major vasoprotective mediator produced by endothelial cells, is primarily synthesized from L‐arginine by endothelial nitric oxide synthase (eNOS), with tetrahydrobiopterin (BH4) serving as an essential cofactor [78]. The physiological effect of NO from the endothelium is known to be associated with its anti‐inflammatory and antithrombotic properties, as well as inhibition of proliferation of VSMCs. At the same time, any pathological factors such as oxidative stress or mechanical injuries from stenting are able to severely affect the generation of NO by the endothelium. This results in an increase in VSMCs proliferation and migration and the secretion of inflammatory cytokines, which promote even more oxidative damage [79]. Together, these interrelated pathological alterations contribute to severe endothelial dysfunction.

#### 3.2.3 ROS

Oxidative stress is an important upstream pathological factor that can lead to the onset and progression of endothelial dysfunction. In normal circumstances, the production of ROS in endothelial cells occurs in very low amounts through three mechanisms, including mitochondria, NADPH oxidases (NOX), and xanthine oxidase (XO) [80]. The intracellular antioxidant defense system controls ROS levels in order to maintain the proper endothelial function. Nevertheless, when the pathological factors are present, the balance between oxidative and antioxidative molecules is disturbed, causing the development of oxidative stress in the blood vessels [81]. On the one hand, the excessive ROS decreases eNOS activity, which leads to reduced production of NO as a vasoprotective agent. On the other hand, elevated ROS activate mitochondrial oxidative damage pathways and the NLRP3 inflammasome, inducing endothelial apoptosis and pyroptosis, respectively, and ultimately leading to endothelial denudation and vascular injury.

In summary, endothelial dysfunction acts as both an initiator and a major driver of neointimal hyperplasia. Specifically, it promotes neointimal formation by disrupting vascular homeostasis, releasing pro‐activation signals, and remodeling the local microenvironment. Therapeutic strategies aimed at preserving endothelial cell function, including inhibition of EndoMT, enhancement of NO signaling, and maintenance of ROS homeostasis, therefore hold considerable potential for the prevention and treatment of neointimal hyperplasia.

### 3.3 Inflammatory Reaction Induced by Elevated Immune Cell Populations

During the progression of pathological neointimal hyperplasia, VSMC phenotypic transition and endothelial dysfunction are major contributors. Meanwhile, the inflammatory microenvironment established through immune cell participation also exerts an important regulatory role (Figure 3). During the early stage of vascular injury, neutrophils are the first immune cells recruited to the lesion. By releasing ROS and neutrophil extracellular traps (NETs), neutrophils directly exacerbate endothelial oxidative damage and compromise endothelial barrier integrity [82]. As vascular injury progresses, circulating monocytes are recruited into the subendothelial space, where they differentiate into M1 and M2 macrophages and continuously regulate VSMC phenotypic transition and proliferative activity.

**FIGURE 3:**
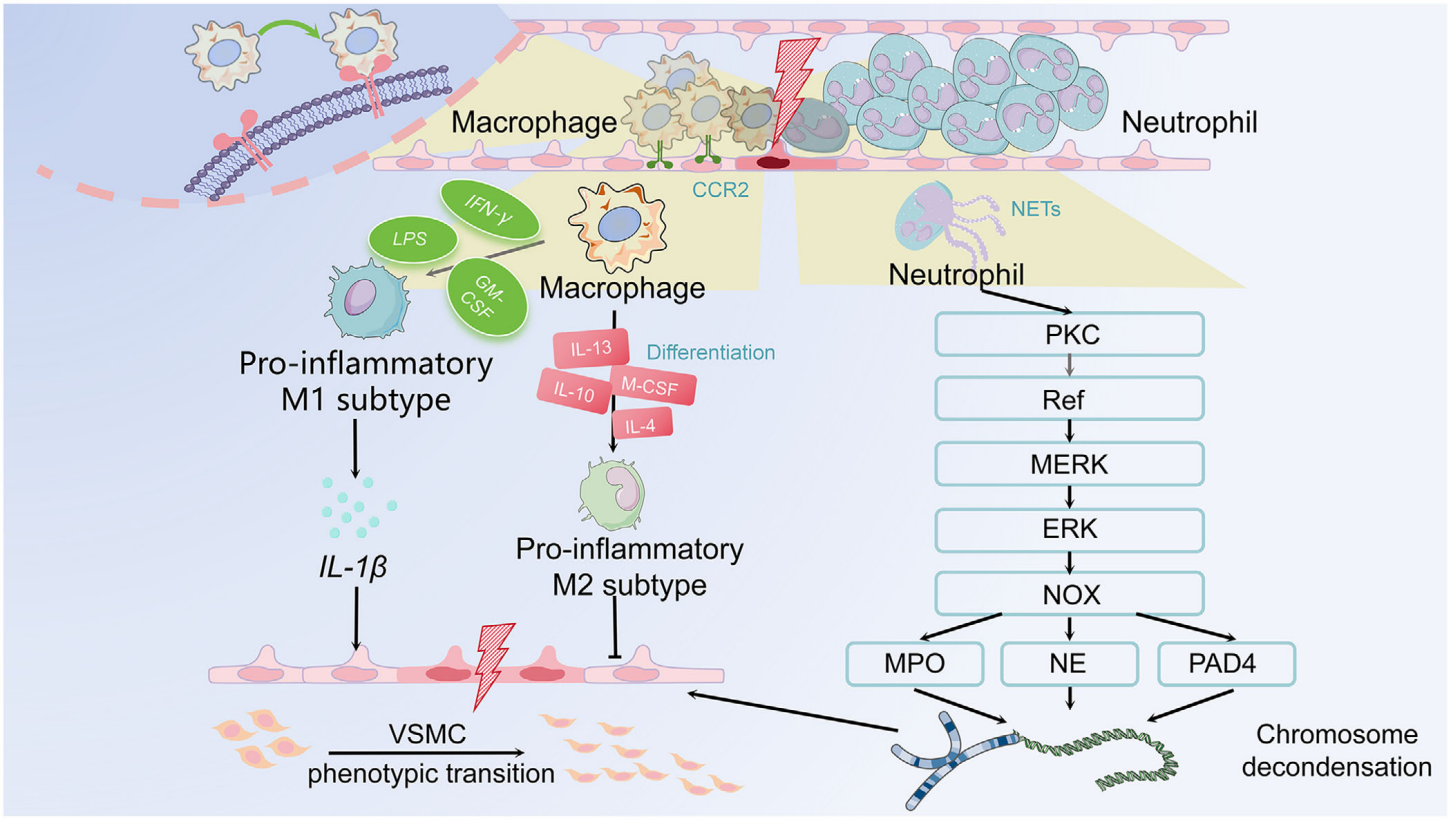
Immune cell‐mediated mechanisms in neointimal hyperplasia. Macrophages differentiate into M1 (pro‐inflammatory) or M2 (anti‐inflammatory) subtypes under the influence of local cytokines. M1 macrophages secrete pro‐inflammatory mediators (e.g., IL‐1β) that induce VSMC phenotypic transition (from contractile to synthetic), promoting proliferation and migration. M2 macrophages inhibit this process by releasing anti‐inflammatory factors, reducing VSMC remodeling. Neutrophils release NETs, which activate the PKC‐Raf/MERK/ERK pathway to stimulate NOX. Activated NOX triggers MPO, NE, and PAD4 activation, leading to chromatin decondensation and increased vascular inflammation, ultimately exacerbating neointimal thickening and vascular stenosis.

### 3.4 Programmed Cell Death

Programmed cell death is an orderly, autonomous process tightly regulated by genetic programs. In recent years, accumulating evidence has demonstrated bidirectional interactions, rather than unidirectional regulation, between different forms of programmed cell death and neointimal hyperplasia [83, 84]. Accordingly, this section systematically summarizes the major programmed cell death mechanisms associated with neointimal hyperplasia, providing a theoretical basis for the development of targeted therapies for vascular injury‐related diseases.

#### 3.4.1 Apoptosis

Apoptosis, the most extensively characterized form of PCD, is a fundamental process that maintains tissue homeostasis by eliminating abnormal or superfluous cells [85]. There are two major pathways of apoptosis, namely the intrinsic or mitochondria‐dependent pathway and the extrinsic or death receptor‐dependent pathway that end in the activation of the caspase cascade resulting in cell degradation. The intrinsic pathway of apoptosis induced by mitochondria is triggered by a variety of signals such as ROS, mechanical stress, and DNA damage. All these stimuli lead to permeabilization of the mitochondria and eventually the release of cytochrome c from the mitochondria to the cytoplasm. Cytochrome c interacts with the Apaf‐1 protein and forms the apoptosome, which results in activation of caspase‐9. Activation of caspase‐9 further leads to the activation of caspase‐3 and caspase‐7, which results in apoptosis. The extrinsic pathway of apoptosis is triggered by the extracellular death ligands. Inflammatory mediators such as TNF‐α and FasL bind to death receptors, including TNFR1 and Fas, on the cell membrane, inducing receptor oligomerization, recruitment of the adaptor protein FADD, and formation of the death‐inducing signaling complex (DISC), which activates the initiator caspase‐8. Activated caspase‐8 regulates downstream signaling through two routes: it can directly cleave and activate executioner caspases‐3/7 to rapidly induce apoptosis or cleave Bid into an active fragment that engages the mitochondrial apoptotic pathway and amplifies apoptotic signaling.

Under physiological conditions, apoptosis selectively eliminates excessive synthetic VSMCs and reduces the population of “pro‐remodeling cells” indirectly favoring the maintenance or restoration of the contractile phenotype in the remaining VSMCs. Under pathological conditions, however, multiple molecular pathways inhibit mitochondrial‐ or death receptor‐mediated apoptosis in VSMCs, thereby promoting neointimal hyperplasia. Previous studies have shown that PDGF‐BB, a member of the platelet‐derived growth factor family that promotes VSMC phenotypic transition, upregulates glucose‐6‐phosphate dehydrogenase (G6PD). G6PD binds to mitochondrial voltage‐dependent anion channel 1 (VDAC1) and competitively interferes with the interaction between Bax and VDAC1, thereby markedly suppressing VSMC apoptosis [83, 86]. In addition, a vascular injury model in Nrf2‐knockout mice indicated that the Nrf2/Keap1 system can modulate VSMC apoptosis by suppressing oxidative stress, thereby reducing neointimal hyperplasia [87].

Notably, in addition to its role in VSMC phenotypic transition, apoptosis is closely associated with endothelial dysfunction. Accumulating evidence has shown that mitochondrial dysfunction upregulates PCSK9 expression, which subsequently reduces SIRT4 expression [88]. As a mitochondria‐protective protein, SIRT4 limits oxidative stress and NF‐κB pathway activation; thus, loss of SIRT4 exacerbates both processes [89, 90]. This signaling cascade ultimately promotes local vascular inflammation and endothelial injury. In addition, endothelial apoptosis causes substantial loss of anticoagulant molecules from the endothelial surface, thereby shifting the vascular wall toward a procoagulant phenotype [91].

#### 3.4.2 Autophagy

Autophagy is an important homeostatic pathway that can also contribute to programmed cell death under specific conditions. It mediates lysosome‐dependent degradation and recycling of damaged cellular components [92]. In mammalian cells, autophagy is principally classified into three subtypes: macroautophagy, microautophagy, and chaperone‐mediated autophagy [93]. Under physiological conditions, autophagy efficiently removes damaged or harmful intracellular components to maintain normal cellular function. Under pathological conditions, however, excessive autophagy may be induced by exogenous stimuli and subsequently contribute to cellular injury [94].

Accumulating evidence indicates that autophagy exerts a dual, context‐dependent effect on the progression of neointimal hyperplasia. On the one hand, moderate autophagy maintains the contractile phenotype of VSMCs and suppresses abnormal vascular proliferation [95]. On the other hand, previous studies have shown that PDGF‐BB can upregulate Beclin‐1‐mediated autophagy through the mTOR pathway [96]. Moreover, vascular injury reduces myocardin expression, leading to subsequent downregulation of miR‐30a, which relieves its inhibitory effect on Beclin‐1 and consequently induces aberrant autophagy activation. Excessive autophagy further decreases the expression of contractile markers, including α‐SMA, SM22α, and calponin, while increasing synthetic markers, thereby enhancing VSMC proliferation and migration [25, 97].

#### 3.4.3 Pyroptosis

Pyroptosis is a form of pro‐inflammatory PCD that has attracted increasing attention in recent years. Unlike relatively immunologically silent cell death, pyroptosis is characterized by cell membrane rupture and extensive release of inflammatory mediators, such as IL‐1β and IL‐18, thereby eliciting a potent inflammatory response [98]. Pyroptosis is primarily regulated through two signaling cascades: the canonical and non‐canonical pathways. The canonical pyroptotic pathway is initiated by the assembly and activation of the NLRP3 inflammasome. Upon activation, the inflammasome stimulates autocleavage and activation of caspase‐1 that leads to subsequent proteolytic cleavage of gasdermin‐D (GSDMD) serving as one of the executors of pyroptosis. Translocation of N‐terminus of GSDMD protein to plasma membrane and formation of membrane pores allows secretion of pro‐inflammatory cytokines [99]. In contrast, the non‐canonical pyroptotic pathway has been mainly investigated in the context of bacterial infections and vascular endotoxemia [100, 101]. This pathway involves human caspases‐4/5 or murine caspase‐11 that cleave GSDMD and induce pyroptosis without participation in canonical inflammasome assembly [102]. It is noteworthy that one of the effectors that can induce pyroptosis includes gasdermin E (GSDME). The mechanism involved here involves activation of caspase‐3, which will then cleave GSDME into GSDME‐N pores that will induce pyroptosis without the involvement of inflammasome or caspase‐4/5/11 [103]. However, evidence regarding GSDME‐associated pyroptosis in neointimal hyperplasia remains limited, and further studies are needed to clarify its potential role in VSMC phenotypic transition.

A vicious loop has been established between pyroptosis and VSMC phenotypic transition. Pyroptosis stimulation causes production of proinflammatory cytokines by the damaged cells, which in turn, through their interaction with specific receptors in VSMCs, induce the inhibition of contraction‐specific marker genes like α‐SMA due to NF‐κB pathway activation [104]. Consequently, the VSMCs that have already undergone phenotypic transition exhibit sustained upregulation of pyroptosis‐associated genes such as NLRP3, caspase‐1, and GSDMD [105]. Moreover, increased intracellular ROS level is considered an appropriate setting for pyroptosis stimulation in VSMCs [106]. Traditionally, pyroptosis has been considered a cell autonomous type of death limited only to those cells that were directly damaged. Recent findings have demonstrated that extracellular vesicles derived from pyroptotic cells contain fully assembled and active pores of GSDMD in their membranes. As a consequence of the fusion of such vesicles with neighboring cells, these pores get transferred to the neighboring cells, thereby leading to their pyroptosis and causing additional damage to the vasculature [107]. This finding expands the mechanistic framework through which pyroptosis may contribute to vascular disorders characterized by pathological neointimal hyperplasia.

#### 3.4.4 Necroptosis

Necroptosis is an inflammatory form of programmed cell death that occurs independently of caspase activity and is primarily regulated by the RIPK1/RIPK3/MLKL signaling axis [108]. Unlike pyroptosis, which can provoke intense local vascular inflammation, necroptosis does not directly promote the maturation of the key pro‐inflammatory cytokines IL‐1β and IL‐18 because it lacks caspase‐1 activity. Instead, its pro‐inflammatory effects are mainly mediated by the release of damage‐associated molecular patterns during cell lysis, which further amplifies local inflammation [109]. Accordingly, necroptosis may function as a complementary inflammatory pathway that acts in concert with pyroptosis.

Following vascular stent implantation, both endothelial cells and VSMCs are susceptible to RIPK3‐dependent necroptosis. Necroptosis of endothelial cells compromises vascular barrier integrity and facilitates the subendothelial infiltration of monocytes and lipids. In contrast, VSMC necroptosis activates NF‐κB‐mediated inflammatory signaling, promoting the phenotypic transition of VSMCs from a contractile to a synthetic state and enhancing their proliferative, migratory, and extracellular matrix‐producing capacities. Multiple animal models of arterial injury have demonstrated that genetic deletion of RIPK3 or pharmacological inhibition of necroptosis with Nec‐1 effectively suppresses VSMC necroptosis and markedly attenuates pathological neointimal thickening after arterial injury.

#### 3.4.5 Ferroptosis

In 2012, Dixon et al. formally identified and named ferroptosis as a novel form of programmed cell death driven by iron overload and lipid peroxidation. Its hallmark is the excessive accumulation of intracellular lipid ROS, which causes oxidative damage and ultimately leads to cell demise [110]. The cystine/glutamate antiporter (System X_c_^−^)‐glutathione (GSH)‐glutathione peroxidase 4 (GPX4) axis constitutes the central antioxidant pathway regulating ferroptosis. This pathway efficiently suppresses lipid peroxidation and is essential for preventing ferroptosis. When GSH is depleted or GPX4 activity is inhibited, intracellular lipid peroxides progressively accumulate and ultimately trigger ferroptosis [111]. In addition, three alternative pathways, namely the FSP1‐CoQ_10_ pathway [112], the GCH1‐BH4 pathway [113], and the dihydroorotate dehydrogenase (DHODH) pathway [114], also contribute to ferroptosis regulation. Mouse carotid artery ligation experiments demonstrate that the abnormal activation of ferroptosis leads to the progression of pathological intimal hyperplasia. In terms of mechanism, the presence of oxidative stress and ROS in ferroptosis stimulates dedifferentiation of the VSMC, resulting in an increase in the proliferation and migration of VSMC [115]. The major enzymatic inhibitor of ferroptosis is GPX4, which reduces lipid hydroperoxides in membranes to lipid alcohols through its activity in the presence of glutathione. However, GPX4 does not decrease ROS in the cytosol, while lipids hydroperoxides cause mitochondrial dysfunction and ROS generation, resulting in redox stress [116]. The sublethal oxidative stress due to production of ROS and lipid hydroperoxides disrupts mitochondrial homeostasis and induces dedifferentiation of VSMCs. The overexpression of GPX4 is known to reduce lipid peroxide‐driven ROS amplification, thus hindering the dedifferentiation process of VSMCs [117]. Macrophages represent important immune cells in the vascular injury microenvironment. Many studies have shown that the phenomenon of ferroptosis in macrophages might play an important role in the pathological development of neointimal hyperplasia due to increased production of inflammatory cytokines such as TNF‐α and IL‐6 [118]. Despite animal and cell‐based studies providing evidence for the role of ferroptosis in pathologic intimal hyperplasia following vascular damage, several issues remain to be solved. First, many studies are performed using small molecules like Fer‐1 and RSL3 that can have off‐target actions. Furthermore, relatively few in vivo studies have used conditional knockout or overexpression of vascular‐specific core molecules such as GPX4 and SLC7A11, limiting the establishment of a direct causal relationship between ferroptosis and neointimal hyperplasia.

#### 3.4.6 Cuproptosis

Cuproptosis is a recently identified form of programmed cell death triggered by intracellular free copper overload. It primarily targets mitochondrial metabolism and is initiated by copper binding to lipoylated tricarboxylic acid (TCA) cycle proteins [119]. Disruption of copper homeostasis causes excessive accumulation of free intracellular copper. Ferredoxin 1 (FDX1), a key mediator of cuproptosis, reduces Cu^2+^ to reactive Cu^+^, which selectively binds to lipoylated TCA enzymes, including dihydrolipoamide S‐acetyltransferase (DLAT) and dihydrolipoamide S‐succinyltransferase (DLST). Sustained copper binding alters protein conformation, induces protein oligomerization and irreversible aggregation, disrupts the TCA cycle, and impairs cellular energy metabolism. Meanwhile, excess copper promotes the loss of iron‐sulfur (Fe‐S) cluster proteins, impairs mitochondrial respiratory chain assembly, and increases ROS production. These combined mitochondrial defects culminate in metabolic collapse and ultimately cuproptosis.

Cuproptosis, being a copper‐mediated pathway of cell death, could be involved in pathological intimal hyperplasia via multiple pathways. Mechanical stress due to stent placement and balloon injury could alter copper metabolism in vascular tissue. ATOX1, the copper transporter protein, increases after vascular injury, and evidence obtained using drugs demonstrates copper imbalance involvement in neointimal hyperplasia, thus, the injured vascular environment could lead to copper imbalance in VSMCs [120, 121]. Excessive copper causes mitochondrial metabolic impairment and excessive ROS formation and stimulates the change of VSMC phenotype from contractile to synthetic, increasing proliferation and migration [122]. Studies involving animals have demonstrated that copper chelators can reduce neointimal hyperplasia caused by injury [121]. Nevertheless, in vivo functional studies targeting core cuproptosis mediators, including FDX1 and lipoylated DLAT, remain limited, and the precise mechanisms by which cuproptosis regulates neointimal hyperplasia require further investigation.

Collectively, there is no sole mechanism in play in VSMC phenotypic transition. Instead, it is due to the combined effect of various regulating factors. While increasing knowledge exists regarding the way different signaling pathways regulate VSMC phenotypic transition, the interaction between these pathways and their regulatory patterns within the physiological environment have yet to be completely understood. Elaborating on the regulatory mechanisms involved in VSMC phenotypic transition can provide insight into how to block inappropriate growth and migration mechanisms to prevent neointimal hyperplasia.

## 4 Animal Models

Neointimal hyperplasia remains a major challenge in cardiovascular health. A deeper understanding of its underlying pathological mechanisms may provide new insights into clinical diagnosis and therapeutic intervention. This section summarizes commonly used animal models of neointimal hyperplasia and discusses their associated molecular regulatory mechanisms to provide a reference for future studies.

### 4.1 Rodent Models

Rodent models, including mice and rats, are the most commonly used experimental animals to study neointimal hyperplasia following vascular injury. Rodents have several benefits, such as lower cost of maintenance, reduced time span of experiments, and well‐defined surgical approaches. Mice have advanced genetic manipulation ability through knockout, overexpression, and conditional approaches and hence can be used for validating the mechanism. On the other hand, rats, owing to their large vasculature, are ideal for interventional studies involving localized drug administration and vector transfection.

#### 4.1.1 Femoral Artery Wire Injury Model

Despite the advent of new animal models, the femoral artery wire injury in mice is still one of the most popular models to study the process of neointima formation following vascular injury and ISR mechanisms. Previously, wire injury has mainly been performed on the femoral artery, but recently, there have been many studies focusing on carotid artery wire injury for the purpose of studying restenosis. The advantages of using the carotid artery include easy access to the vessel as well as better simulation of clinical vascular injury [123]. For model construction, the animals are put under anesthesia, and a unilateral femoral artery is isolated. A blunted metal wire is then inserted retrogradely through the vascular lumen via a puncture made on the distal segment of the femoral artery. Friction induced to the inner surface of the vessel causes mechanical injury by abrading off the endothelial lining. The procedure creates mechanical injury to the vascular wall [124], leading to a breach in the integrity of the endothelium, which further leads to phenotypic transition of VSMCs from the contractile to synthetic mode and their proliferation and migration towards the lumen, thus forming a neointima. Despite having a well‐documented methodology, the method is less efficient compared to other methods in constructing models of smaller luminal vessels.

#### 4.1.2 Carotid Artery Ligation Model

The carotid artery ligation model differs from models with endothelial damage caused by direct mechanical injury in that the reduction in carotid blood flow and shear stress causes endothelial dysfunction. The model is achieved with the help of anesthesia in the form of isoflurane inhalation (4% for anesthesia induction and 1.5%–2% for maintenance). An anterior cervical incision is made, followed by the blunt separation of the left common carotid artery between the sternohyoid muscles. The procedure is carried out with the utmost care to avoid injuring the vagus nerve and overstretching of vessels that can cause unintended injury to the endothelium. Ligation is performed above the carotid bifurcation with the help of sterile 8‐0 silk suture. Once complete obstruction of the blood flow is confirmed, the incision is closed layer‐by‐layer. The tissue samples are obtained 4 weeks after surgery [125, 126]. The C57BL/6J mouse is considered to be the main strain used in preclinical studies utilizing the carotid artery ligation approach [127, 128]. This strain is characterized by the formation of neointimal lesions under the effect of altered hemodynamics; therefore, the results obtained can be predicted. Moreover, C57BL/6J mice exhibit favorable surgical tolerance and stable postoperative survival after carotid ligation, facilitating subsequent mechanistic and interventional studies [129].

#### 4.1.3 Carotid Artery Balloon Injury Model

Carotid artery balloon injury is another widely used animal model for investigating ISR and primarily promotes neointimal hyperplasia by driving the phenotypic transition of VSMCs from a contractile to a synthetic state [130]. To establish the model, rats are anesthetized and the left common carotid artery is exposed. A small incision is made in the external carotid artery, through which an inflatable balloon catheter is advanced retrogradely into the common carotid artery. After low‐pressure inflation, the catheter is slowly withdrawn, and this procedure is repeated several times to achieve complete endothelial denudation. After catheter removal, the proximal external carotid artery is ligated, and the incision is closed in layers. Tissue samples are typically collected 14–21 days after surgery [131].

### 4.2 Rabbit Model

Although mouse models offer advantages such as low cost, genetically manipulable backgrounds, and short experimental cycles, rabbit models are also widely used because they more closely recapitulate the pathological features of human ISR. Among these, New Zealand White rabbits are the most commonly used strain [132]. Compared with rodents, rabbits have larger arterial lumens, permitting endovascular stent implantation to mimic the mechanical vascular injury caused by clinical coronary stenting. Currently, two major models are commonly used: the rabbit carotid artery stent implantation model and the rabbit iliac artery balloon endothelial injury model [133, 134]. Nevertheless, their broader application in molecular mechanistic studies is limited by higher experimental costs, longer breeding and experimental cycles, and the limited availability of genetically modified strains [135].

In summary, animal models are indispensable tools for investigating neointimal hyperplasia. Table 4 summarizes the principal therapeutic strategies and associated mechanisms targeting neointimal hyperplasia across different preclinical animal models and may provide a reference for future translational studies of therapeutic targets for neointimal hyperplasia.

**TABLE 4:** Summary of therapeutic strategies and related mechanisms regulating neointimal hyperplasia in preclinical animal models.

Intervention factors/programs	Animal model	Key signaling pathways	Specific mechanisms	Reference
SGLT2 antagonist (empagliflozin)	Carotid balloon injury in rats	PDGF/Akt/STAT3	Inhibition of PDGF‐induced VSMC proliferation and migration	[136]
Colchicine	Carotid artery ligation injury in mice	FOXO3A/SRF‐MYOCD axis, and FOXO3A‐mediated autophagy pathway	Stabilization of VSMC contractile phenotype; suppression of monocyte‐macrophage recruitment and inflammatory cascades	[137]
Vasostatin‐2	Steel wire injury to the femoral artery in mice	ACE2/MasR/PPARγ/NR1D1/Gas1 axis	Inhibition of VSMC phenotypic transition from contractile to synthetic after injury	[138]
FPR2 agonist (BMS‐986235)	Steel wire injury to the femoral artery in mice	FPR2/ELOVL6 axis	Restraining pathological M2 macrophage polarization and sustaining the contractile phenotype of VSMCs	[139]
Inflachromene	Steel wire injury to the carotid artery in mice	TLR4/NF‐κB axis	Inhibition of Ang II‐induced phenotypic transition, proliferation, and migration of VSMCs	[140]
Statins (Rosuvastatin)	Abdominal aorta balloon injury in rabbits	Akt/mTOR/NF‐κB/STAT3 axis	Suppression of pro‐inflammatory factor production and inhibition of VSMC proliferation and migration	[141]

## 5 Clinical Manifestation

An investigation of patients undergoing coronary interventions in China showed that PCI is among the most commonly performed interventional procedures for ischemic heart disease [142]. The widespread adoption of PCI began in the late 1970s with advances in angioplasty techniques [143]. Nevertheless, as discussed above, endothelial injury during PCI induces local inflammatory responses and excessive VSMC proliferation, thereby promoting neointimal hyperplasia, a major precursor of ISR. Although advances such as DES have markedly reduced the incidence of restenosis, they have not eliminated it, and ISR remains a major limitation of PCI. Consequently, reducing the risk of post‐PCI restenosis remains a major clinical challenge. In addition, neointimal hyperplasia contributes to vein graft failure and neoatherosclerosis, further complicating the management of cardiovascular disease.

### 5.1 In‐Stent Restenosis (ISR)

ISR refers to the progressive re‐narrowing of a coronary artery at or adjacent to the site of stent implantation. This pathological process may begin within days after stenting and continue for months or even years, compromising the long‐term clinical benefits of PCI [144, 145]. Angiographically, ISR is typically defined as a reduction in lumen diameter of >50% within the stent or within 5 mm of either stent edge [146].

The pathogenesis of ISR is multifactorial. With the aid of intravascular imaging, its underlying mechanisms can generally be classified into mechanical and biological factors [147, 148]. Mechanical causes include stent underexpansion and stent fracture [149]. In the absence of mechanical abnormalities, neointimal formation represents a major driver of ISR [150]. Non‐mechanical ISR is also associated with complex or prolonged PCIs, with inflammatory mediators and ROS acting as important biological triggers [151, 152, 153]. Although DES release antiproliferative agents to suppress cellular proliferation and reduce ISR, DES‐associated restenosis still occurs, most commonly within 6–9 months after implantation. This phenomenon has been associated with inflammatory and hypersensitivity reactions to the metallic alloys and polymers used in DES [154, 155].

### 5.2 Neoatherosclerosis

Atherosclerosis is the principal pathological basis of atherosclerotic cardiovascular diseases (ASCVDs), for which PCI represents an important interventional treatment [156]. Previous studies examining the relationship between atherosclerosis and neointimal hyperplasia have largely regarded neointimal hyperplasia as a complication of interventions used to treat atherosclerotic lesions. More recent evidence, however, suggests a reciprocal relationship between these two pathological processes. Nakazawa et al. reported the consistent presence of foamy macrophages within different types of implanted vascular stents, a finding closely associated with the development of neoatherosclerosis [157]. Early pathological studies of post‐stenting neoatherosclerosis likewise demonstrated substantial accumulation of foamy macrophages within the neointima, with their formation appearing independent of the organization of the pre‐existing atherosclerotic lesion [158]. VSMC phenotypic transition is accompanied by the production of multiple inflammatory mediators that promote local inflammation. In parallel, the neointima facilitates macrophage infiltration, thereby contributing to the development and progression of neoatherosclerosis [159].

### 5.3 Vein Graft Failure

Coronary artery bypass grafting (CABG) remains an important therapeutic option for patients with complex multivessel CAD [160]. Saphenous vein grafts (SVGs) are commonly used as conduits in CABG; however, graft failure remains frequent. Approximately 8%–25% of SVGs become occluded within the first year after surgery, and only 50%–60% remain patent at 10 years [161]. Neointimal hyperplasia is a major pathological contributor to vein graft failure following CABG [162].

Venous neointimal hyperplasia provides the pathological substrate for subsequent SVG atherosclerosis. Compared with atherosclerosis in native coronary arteries, SVG atherosclerosis progresses more rapidly. The process begins with the accumulation of foam cells within the graft wall, followed by gradual formation of a necrotic core. These changes commonly develop within 5 years after surgery, after which the necrotic core may progress to an atherosclerotic plaque. Subsequent plaque rupture and thrombosis are major causes of graft occlusion [161].

## 6 Diagnosis

Neointimal hyperplasia is a hallmark pathological feature of ISR. On clinical imaging, ISR is considered clinically significant when diameter stenosis exceeds 50% in the presence of ischemic symptoms or objective evidence of ischemia, or exceeds 70% irrespective of ischemic manifestations [163]. Imaging therefore plays a central role in evaluating neointimal hyperplasia. This section summarizes the principal imaging modalities used for its assessment.

### 6.1 Coronary Angiography

CAG is the conventional invasive modality for diagnosing ISR [164]. Following intracoronary contrast administration, luminal stenosis can be visually assessed and quantitatively evaluated using quantitative coronary angiography (QCA). However, CAG depicts only the luminal contour and does not directly visualize the vessel wall; therefore, it cannot directly quantify neointimal hyperplasia. Moreover, as an invasive procedure, it carries procedural risks and may cause additional endothelial injury, limiting its suitability for repeated assessment [163].

### 6.2 Intravascular Ultrasound

IVUS is an invasive intracoronary imaging modality that provides real‐time cross‐sectional visualization of the vessel [165]. By advancing an ultrasound catheter into the target vessel, IVUS enables assessment of the vascular wall, including the intima, media, adventitia, and implanted stent structure [166]. The severity of neointimal hyperplasia can be quantitatively assessed primarily by measuring the neointimal area [167].

Compared with CAG, IVUS enables visualization of the entire vessel wall and quantitative measurement of stent and neointimal areas, thereby allowing objective assessment of neointimal hyperplasia and facilitating the diagnosis of ISR. Nevertheless, its moderate spatial resolution limits the precise characterization of subtle neointimal components, including lipid deposits, fibrous tissue, and microcalcifications. Technological advances incorporating higher‐frequency transducers may improve intravascular spatial resolution and enable more accurate differentiation of neointimal tissue phenotypes [168]. In addition, artificial intelligence‐based algorithms may reduce operator‐dependent measurement variability and facilitate real‐time quantitative analysis during catheterization [169].

### 6.3 Optical Coherent Tomography

OCT is widely used to visualize the internal structure of coronary arteries. Similar to IVUS, OCT provides detailed cross‐sectional images of the vessel, facilitating accurate assessment of coronary lesions [170]. Compared with IVUS, OCT offers substantially higher spatial resolution, enabling quantitative measurement of neointimal thickness and differentiation of distinct neointimal phenotypes, thereby overcoming a major limitation of IVUS in tissue characterization [171]. Nevertheless, OCT has inherent limitations. The limited penetration depth of near‐infrared light restricts visualization of deeper vascular wall structures. In addition, temporary clearance of blood from the target vessel segment is required during image acquisition, increasing procedural complexity [172].

### 6.4 Non‐Invasive Imaging Diagnosis

Unlike invasive intracoronary imaging, non‐invasive modalities avoid catheter‐related procedural risks and are widely used for screening and long‐term surveillance of suspected ISR. Coronary computed tomography angiography (CCTA) is the most commonly used non‐invasive anatomical imaging modality and enables three‐dimensional reconstruction of coronary stents and luminal morphology for ISR detection [173]. However, CCTA has important limitations. Image quality is affected by metal blooming artifacts from stent struts, and CCTA cannot quantify neointimal burden or distinguish neointimal tissue phenotypes, restricting its role primarily to the initial evaluation of suspected ISR. Non‐invasive stress myocardial imaging modalities include stress cardiac magnetic resonance (CMR), SPECT, PET, and stress echocardiography. These techniques detect stress‐induced myocardial ischemia and help determine whether ISR lesions are hemodynamically significant. However, they cannot provide morphological information on neointimal hyperplasia and generally need to be combined with anatomical imaging for comprehensive evaluation [163].

Although imaging examinations are central to confirming ISR and assessing its severity, they should be integrated with other complementary evaluations. A multimodal diagnostic approach may facilitate comprehensive assessment and guide individualized management of patients after PCI.

## 7 Treatment

Neointimal hyperplasia is a form of pathological vascular remodeling that develops following endothelial injury. VSMC phenotypic transition and endothelial dysfunction are major determinants of adverse outcomes after vascular interventions. Local delivery of antiproliferative drugs through DES and systemic pharmacotherapy are the main strategies used to suppress excessive VSMC activation. However, long‐term complications, including late stent thrombosis and systemic drug‐related toxicity, remain important barriers to optimal clinical outcomes. Here, we summarize current clinical strategies and recent advances for managing neointimal hyperplasia, discuss the advantages and limitations of existing therapies, and consider the development and clinical translation of emerging treatment approaches.

### 7.1 Vascular Stent

As discussed above, neointimal hyperplasia, particularly ISR, is a major determinant of clinical outcomes after PCI. Current efforts to improve PCI outcomes have therefore focused substantially on optimizing the properties of implanted stents. Clinical performance is influenced by several factors, including stent design, material composition, strut thickness, and vessel wall coverage [154]. This review discusses strategies involving stent optimization and post‐interventional management that may improve the prevention and treatment of ISR.

#### 7.1.1 Introduction of Vascular Stent

PCI uses cardiac catheterization techniques to restore coronary blood flow and has been performed clinically for approximately four decades. Its early approach was plain old balloon angioplasty (POBA), which enlarges the coronary lumen through balloon dilation. However, wider clinical application of POBA revealed important limitations. Acute vascular occlusion after balloon angioplasty occurs in approximately 5%–10% of patients [174]. Because of these risks, the use of conventional balloon angioplasty alone subsequently declined. In the 1980s, the introduction of BMS improved clinical outcomes by providing sustained mechanical support to the coronary artery and reducing complications of POBA, including abrupt vessel closure and acute recoil [174]. However, BMS implantation can induce excessive VSMC activation and proliferation, resulting in neointimal hyperplasia [175]. Indeed, ISR occurs in up to 30% of patients treated with BMS.

The introduction of DES represented a major advance in interventional cardiology. Compared with BMS, DES locally release anti‐inflammatory, immunomodulatory, and/or antiproliferative agents at sites of arterial injury to inhibit neointimal hyperplasia. First‐generation DES were introduced in the early 2000s and markedly reduced the incidence of restenosis [176, 177]. However, late stent thrombosis emerged as a major limitation [178]. Second‐generation DES were developed to overcome the shortcomings of first‐generation devices, with clinical data indicating an approximately 70% reduction in late stent thrombosis [179]. Third‐generation DES are currently under development and incorporate further improvements, particularly thinner struts and more biocompatible or bioabsorbable components, with the aim of reducing neointimal hyperplasia and stent thrombosis [180, 181, 182].

Biodegradable vascular scaffolds (BVS) and drug‐eluting balloons (DEB) are two non‐metallic interventional approaches for coronary artery disease [183]. Owing to their biocompatibility and biodegradability, BVS were developed to potentially reduce endothelial dysfunction, restenosis, and complications associated with permanent metallic implants. However, relatively thick BVS struts, reaching approximately 170 µm, may increase vascular injury and thrombotic risk [184, 185]. Furthermore, angiographic and OCT studies have shown that late scaffold disintegration after implantation may promote neointimal hyperplasia, posing an additional clinical concern [186, 187]. Notably, the first‐generation absorbable BVS was withdrawn from the global market largely because of a higher risk of scaffold thrombosis than conventional metallic DES, highlighting the challenges associated with the clinical translation of biodegradable vascular devices [188, 189]. DEB consist mainly of therapeutic agents, such as paclitaxel, excipients such as butyryl‐tri‐hexyl citrate, shellac, polyethylene glycol, and resveratrol, and balloon platforms [190, 191]. The procedure generally involves lesion preparation with a standard or high‐pressure balloon, followed by local delivery of the antiproliferative drug to suppress neointimal growth [192]. Compared with DES, DEB avoid permanent metallic implantation, provide uniform drug delivery to the vessel wall, and may reduce the risks associated with permanent stents [193, 194, 195]. These characteristics make DEB an attractive option for the treatment of CAD, particularly ISR. A meta‐analysis of ten randomized trials comparing DEB angioplasty with DES implantation for ISR found that DEB produced better outcomes than first‐generation DES, whereas this advantage was not observed when DEB were compared with second‐generation DES. Thus, the relative efficacy of DEB and DES for ISR depends partly on the generation of DES used for comparison [196]. Nevertheless, DEB treatment may cause intimal dissection, which can lead to vessel occlusion and potentially interfere with vascular healing [197].

#### 7.1.2 Stent Selection

The choice of stent is a major determinant of PCI outcomes. Stent implantation commonly induces inflammatory responses within the arterial wall, which may promote neointimal hyperplasia and ultimately restenosis. Appropriate stent selection is therefore important for minimizing these complications and improving the long‐term efficacy of PCI.

Several studies have sought to improve therapeutic efficacy through the modification of metallic stent platforms. From a physiological perspective, inflammation is not terminated simply by depletion of inflammatory cells or mediators but is actively resolved through specific signaling pathways [198, 199]. Among the molecules involved in this process, chemerin is an inflammatory mediator belonging to the chemotactic protein superfamily and exerts its biological effects by binding to ChemR23, a G protein‐coupled receptor (GPCR) [200]. The broad expression of ChemR23 enables chemerin and its derived peptides to exert important regulatory effects in inflammatory diseases [201, 202]. Accumulating evidence indicates that chemerin‐derived cleavage products such as chemerin 15 possess anti‐inflammatory activity. Chemerin 15 is a 15‐amino acid peptide derived from the C‐terminal region of chemerin and can modulate macrophage polarization and reduce inflammatory cytokine expression through ChemR23 in models of peripheral blood inflammation [203, 204]. Wen et al. developed a novel vascular stent functionalized with immobilized chemerin 15 peptides to reduce endothelial injury and inflammation and thereby suppress ISR [199]. The C15‐containing coating may limit early endothelial injury, attenuate inflammatory responses, and reduce the risk of stent thrombosis [205, 206].

As discussed above, BVS have considerable potential for PCI; however, their propensity to induce inflammation has limited their routine application. Poly(L‐lactic acid) (PLLA) possesses favorable mechanical properties, biodegradability, and biocompatibility and is therefore the predominant material used for BVS. However, acidic degradation products generated during PLLA hydrolysis lower the local pH and may induce pronounced inflammatory responses, representing a major limitation of PLLA‐based scaffolds [207]. In addition, PLLA has poor radiopacity, making BVS localization difficult using X‐ray computed tomography (CT) [208]. To overcome these limitations, Lee et al. developed a multilayer‐coated PLLA BVS. The primary coating contains 2,3,5‐triiodobenzoic acid (TIBA), magnesium hydroxide (MH), and poly (D, L‐lactic acid) (PDLLA). TIBA enhances scaffold radiopacity, MH buffers local acidity, and PDLLA reduces TIBA‐associated cytotoxicity [209, 210]. The outer PLLA layer incorporates polydopamine (PDA) and cystamine (Cys), which promote NO production and thereby inhibit post‐PCI neointimal hyperplasia [207, 211]. Although these modified stent platforms have shown promising anti‐restenotic effects in preclinical studies, their clinical translation faces substantial regulatory challenges. Stents with bioactive coatings are generally classified as drug‐device combination products. Regulatory pathways vary according to the primary mode of action and differ among agencies such as the US FDA, European Medicines Agency (EMA), and National Medical Products Administration (NMPA) [212]. Market approval requires rigorous evaluation of coating biocompatibility, degradation behavior, drug‐release profiles, and long‐term safety. Moreover, modifications to polymers, drug formulations, or coating structures often necessitate additional testing. The lack of harmonized international standards, together with lengthy regulatory procedures and stringent evidentiary requirements, continues to impede the clinical translation of experimental stent coatings designed to prevent neointimal hyperplasia.

In conclusion, optimal stent materials should combine adequate biocompatibility and biodegradability to accommodate the vascular environment and minimize inflammatory responses, while retaining sufficient radiopacity for postoperative imaging surveillance.

#### 7.1.3 Coating Selection

Stent coatings serve as major platforms for local drug delivery. Through engineered material structures, these systems can provide controlled and localized release of therapeutic agents. Ideally, stent surfaces should exhibit favorable biological properties, including biocompatibility, anti‐inflammatory activity, rapid endothelialization, and antiproliferative effects [213, 214]. Rapamycin and paclitaxel are traditional coating agents widely used to suppress cellular proliferation. However, rapamycin may cause interstitial pneumonia, while neither agent directly regulates redox homeostasis or exhibits strong cellular selectivity for endothelial cells and VSMCs [215, 216, 217].

Late thrombosis remains an important challenge associated with DES. Epigallocatechin‐3‐gallate (EGCG), a bioactive compound derived from green tea, inhibits platelet aggregation and MMP activity and exhibits antioxidant, anti‐inflammatory, and antithrombotic properties [218, 219]. Tanshinone IIA sodium sulfonate (TSS), a water‐soluble derivative of tanshinone IIA, also exerts potent anti‐inflammatory effects and protects against endothelial dysfunction [220, 221]. By combining the properties of these two agents, Wan et al. developed a sandwich‐structured stent coating. Its polyelectrolyte inner layer, composed of 3‐aminophenylboronic acid‐modified hyaluronic acid and carboxychitosan, is loaded with EGCG‐TSS complexes and can suppress thrombosis and ISR [222]. As noted above, NO is an important signaling molecule that maintains endothelial function and inhibits VSMC proliferation [223]. Coatings capable of sustaining NO release at physiological concentrations may therefore suppress neointimal hyperplasia and reduce long‐term ISR. Chen et al. developed a nitric oxide‐eluting (NOE) hydrogel coating composed of alginate and gelatin. This coating provides sustained NO generation while maintaining favorable mechanical properties, thereby reducing post‐procedural restenosis and promoting rapid endothelial recovery [224]. In addition, ebselen exhibits glutathione peroxidase (GPX)‐like activity, enabling hydroperoxide scavenging and regulation of ROS‐associated redox homeostasis. Wang et al. developed a novel coating incorporating ebselen and poly (trimethylene carbonate) (PTMC), which attenuated ROS‐mediated oxidative injury while supporting NO release and maintaining favorable biocompatibility [225]. Overall, NO‐releasing coatings can promote endothelial cell migration and proliferation, suppress excessive VSMC growth, and accelerate re‐endothelialization, indicating considerable translational potential. However, as with other advanced stent platforms, devices incorporating natural phytochemicals, NO donors, or antioxidant agents are generally classified as drug‐device combination products. Their commercialization therefore requires extensive evaluation and approval by different regulatory authorities, which remains a major obstacle to clinical translation.

#### 7.1.4 Postoperative Recovery

Although PCI plays a central role in determining patient prognosis, appropriate postoperative management is equally important. After PCI, pharmacological therapy is required to prevent complications such as ISR [226]. Currently available agents include anticoagulants, anti‐inflammatory drugs, and antiproliferative agents. However, some of these drugs may also inhibit endothelial cell proliferation and migration, thereby delaying endothelial repair and potentially increasing the risk of ISR [227]. Therefore, promoting re‐endothelialization at sites of vascular injury represents a promising strategy for post‐interventional arterial repair and restenosis prevention [228]. Endothelial progenitor cells (EPCs), as precursors of vascular endothelial cells, play an important role in postoperative vascular recovery. On the one hand, EPCs can differentiate into endothelial cells, thereby promoting re‐endothelialization and reducing the risk of neointimal hyperplasia and stent thrombosis [229]. On the other hand, EPCs secrete vascular endothelial growth factor (VEGF), which further facilitates endothelial regeneration [230].

Two principal strategies can enhance re‐endothelialization: promoting EPC mobilization and homing and improving VEGF‐targeted delivery. Regarding EPC mobilization and homing, studies have demonstrated that neurotrophic molecules can induce EPC mobilization and recruitment, thereby promoting the endothelialization of tissue‐engineered blood vessels [231]. Based on this concept, Chen et al. proposed that neurotrophin‐3 (NT‐3), a specific neurotrophic factor, could accelerate endothelialization of injured vessels by promoting EPC homing [232]. To enhance vascular VEGF targeting, Hao et al. engineered biodegradable and biocompatible gene‐delivery complexes carrying the PCDNA3.1‐VEGF165 plasmid, which markedly increased VEGF expression in vivo [233].

### 7.2 Drug Treatment

#### 7.2.1 Drug Selection

Extensive evidence indicates that multiple signaling pathways in vascular endothelial cells contribute to the development of neointimal hyperplasia [234, 235, 236]. Nevertheless, relatively few studies have clarified how these pathways regulate vascular endothelial cell proliferation and translated these findings into targeted therapeutic strategies. Identifying novel signaling targets and developing specific therapeutic agents therefore remain important research priorities for suppressing the pathogenic mechanisms underlying post‐procedural ISR (Figure 4).

**FIGURE 4:**
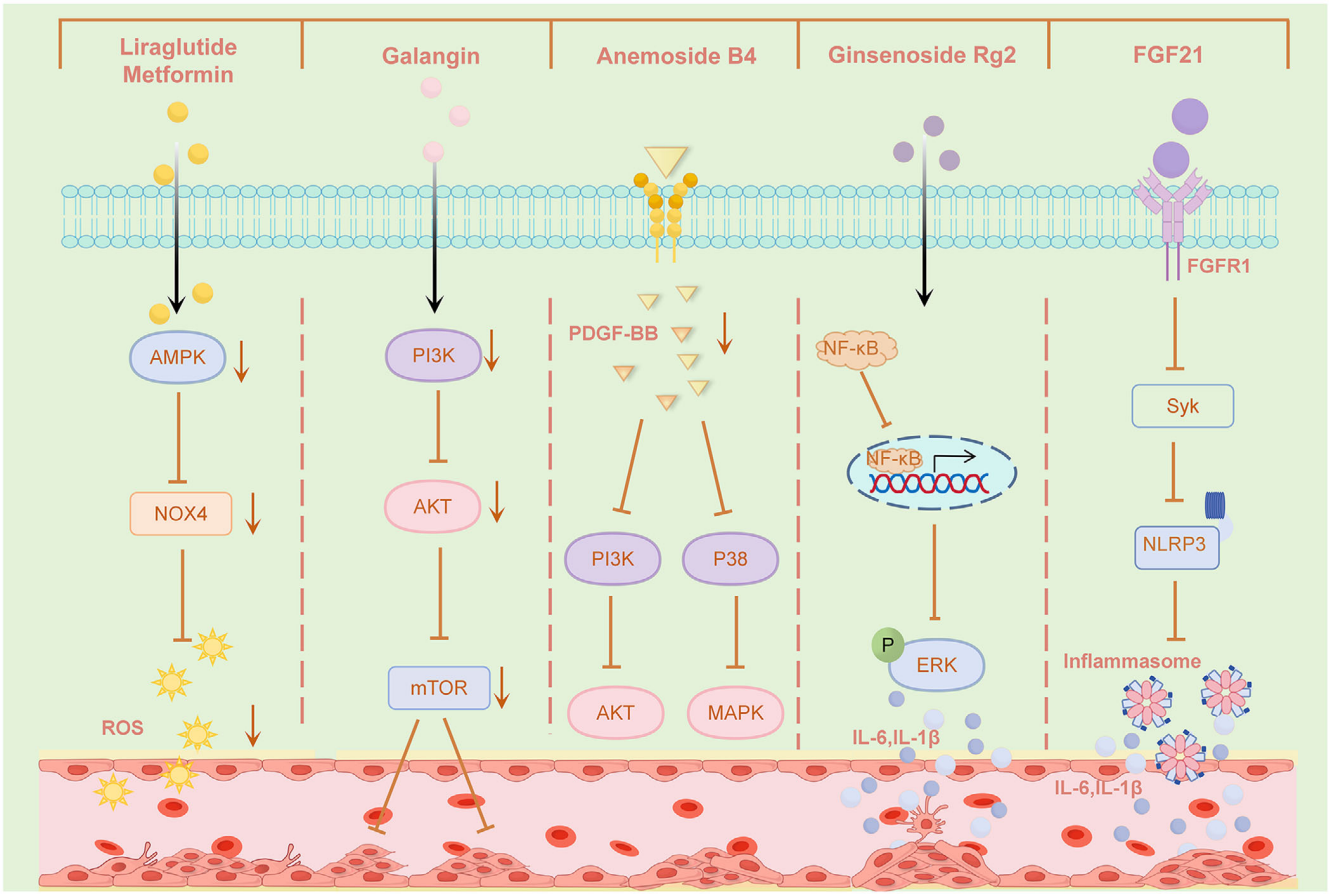
Pharmacotherapies for vascular stenosis. Current treatments primarily target two pathways to alleviate neointimal hyperplasia: reducing the proliferation and migration of VSMCs and mitigating vascular inflammation. Therapies inhibiting VSMC proliferation and migration include combination therapy with linagliptin and metformin, as well as galangin and Anemoside B4. Regarding inflammation alleviation, ginsenoside Rg2 and fibroblast growth factor 21 have demonstrated promising efficacy.

Diabetes can increase NADPH oxidase‐derived ROS production, thereby increasing susceptibility to neointimal hyperplasia. In diabetic rat models, the AMP‐activated protein kinase (AMPK)/Nox4 signaling pathway has been implicated in the development of neointimal hyperplasia. AMPK regulates VSMC proliferation and migration, both of which contribute to neointimal lesion formation [237]. Nox4 belongs to the NADPH oxidase family. Although its precise relationship with neointimal hyperplasia remains incompletely defined, several studies have linked Nox4 upregulation to increased ROS production, suggesting that it may represent a potential therapeutic target [238]. Other studies have identified Nox4 as a downstream effector of AMPK [237, 239]. Pathological vascular injury reduces AMPK activity, thereby relieving its inhibitory effect on Nox4 and promoting excessive ROS production in VSMCs. Increased ROS subsequently drives VSMC dedifferentiation, proliferation, and migration, accelerating neointimal hyperplasia. Accordingly, pharmacological restoration of AMPK‐dependent Nox4 suppression may protect against neointimal lesion formation. Zhang et al. demonstrated that combined linagliptin and metformin treatment may exert synergistic effects by modulating the AMPK‐Nox4 axis and thereby suppressing vascular neointimal hyperplasia [239]. Beyond preclinical evidence, a single‐center, parallel‐group, double‐blind randomized clinical trial showed that, in patients with impaired glucose tolerance (IGT) and at least two risk factors for type 2 diabetes (T2D), combined linagliptin and metformin therapy improved glucose tolerance and pancreatic β‐cell function more effectively than metformin monotherapy [240]. However, this trial evaluated only metabolic outcomes and did not directly quantify neointimal hyperplasia. Therefore, whether combination therapy modulates neointimal hyperplasia through improved glycemic control requires further validation using intracoronary imaging modalities such as IVUS or OCT.

To suppress excessive VSMC proliferation and migration, another study showed that galangin, a bioactive compound derived from ginger, modulates the PI3K/AKT/mTOR pathway and thereby inhibits VSMC proliferation and migration [236]. In addition, PDGF‐BB can activate the downstream ROS/NF‐κB/mTOR/P70S6K signaling cascade, promoting VSMC phenotypic transition and consequently contributing to neointimal hyperplasia [29, 241]. Shan et al. found that anemoside B4 (AB4), a bioactive component isolated from Pulsatilla chinensis, inhibited PDGF‐BB‐induced VSMC proliferation and migration. In a murine model of acute femoral artery injury, AB4 attenuated signaling pathways associated with neointimal hyperplasia through modulation of the PI3K/AKT and P38 MAPK pathways [242].

As discussed above, inflammation is another major contributor to neointimal hyperplasia. Targeting inflammatory signaling pathways therefore represents a potential therapeutic strategy. Ginsenoside Rg2, a major bioactive component of ginseng, has been shown to reduce inflammatory mediator expression in human umbilical vein endothelial cells (HUVECs) by inhibiting NF‐κB and p‐ERK signaling, thereby attenuating inflammatory responses [243, 244]. In diabetic mice with common carotid artery (CCA) injury, fibroblast growth factor 21 (FGF21) also exerted anti‐neointimal effects. As an important regulator of glucose metabolism in diabetes, FGF21 suppresses neointimal hyperplasia by modulating the inflammatory FGFR1/Syk/NLRP3 signaling pathway. Furthermore, a clinical study involving patients with T2D demonstrated significantly elevated serum FGF21 levels in individuals with subclinical atherosclerosis [245, 246]. These findings suggest that FGF21 may represent a potential therapeutic target for neointimal hyperplasia, although further strategies to enhance FGF21 responsiveness require investigation.

#### 7.2.2 Drug Delivery System

In addition to identifying novel pharmacological agents based on the mechanisms underlying neointimal hyperplasia, recent research has focused on improving drug‐delivery efficiency to ensure adequate accumulation at target sites. Failure to deliver therapeutic agents selectively and efficiently to the intended vascular lesion can substantially limit their therapeutic efficacy.

Nanotechnology provides promising drug‐delivery platforms for the treatment of neointimal hyperplasia. Nanocarriers are generally defined as particulate systems with dimensions of approximately 1–100 nm. They can overcome several limitations of conventional drug administration by enabling targeted delivery and spatiotemporal control of drug release. Unlike DES, which primarily suppress cellular proliferation, nanoparticle‐based systems may not only reduce drug‐related adverse effects but also modulate inflammation through regulation of macrophage function, thereby attenuating neointimal hyperplasia [247, 248]. Despite encouraging preclinical findings, most nanotechnology‐based therapeutics have not progressed successfully through clinical evaluation because of unresolved safety concerns [249]. Further optimization of formulation design and comprehensive pharmacokinetic assessment are therefore required before nanoparticles can be safely and routinely applied to coronary artery lesions.

Stimuli‐responsive drug delivery systems (SRDDSs) represent an emerging approach to controlled drug delivery. These systems enable precise regulation of drug release in response to specific environmental or physiological stimuli, including temperature, pH, redox conditions, and light exposure [250].

### 7.3 Emerging Therapeutic Approaches

Beyond conventional approaches, including PCI and pharmacological modulation of VSMC activity, recent studies have explored alternative therapeutic strategies such as stem cell therapy and IL‐11 inhibition.

P‐selectin is a member of the selectin family, together with L‐selectin and E‐selectin. Several studies have demonstrated that targeted inhibition of P‐selectin overexpression can ameliorate pathological vascular responses [251, 252]. Accordingly, Yan et al. developed a novel stem cell‐based strategy using adipose‐derived stem cells (ADSCs) modified with a linker composed of a P‐selectin‐binding peptide (PBP) and a polyethylene glycol‐conjugated phospholipid derivative (DMPE‐PEG), termed DMPE‐PEG‐PBP (DPP). This modification enables targeted adhesion to injured arteries and occupies P‐selectin‐binding sites, thereby attenuating P‐selectin‐mediated pathological effects. In addition, ADSCs can reduce the expression of pro‐inflammatory mediators such as IL‐1 and IL‐6, thereby promoting re‐endothelialization and inhibiting VSMC migration [253]. Another study demonstrated that IL‐11 signaling promotes TGF‐β_1_‐ or ANGII‐induced VSMC dedifferentiation in mice, resulting in phenotypic transition [254]. Accordingly, Schumacher et al. administered IL‐11‐targeting antibodies to mice and found that IL‐11 inhibition significantly reduced vascular wall thickening, attenuated neointimal hyperplasia, and promoted favorable plaque remodeling following vascular injury [255].

## 8 Conclusion and Future Perspectives

ISR remains a common complication following PCI, with neointimal hyperplasia representing its principal pathological feature and an important focus of current research. As discussed above, neointimal hyperplasia is regulated by multiple interacting factors and overlapping molecular pathways. Elucidating these underlying mechanisms and their complex interactions is essential for the development of more effective therapeutic strategies.

Accurate assessment of neointimal hyperplasia is critical for monitoring lesion progression after PCI. Intracoronary imaging currently represents the principal approach for visualizing and quantitatively evaluating neointimal hyperplasia. OCT and IVUS enable cross‐sectional assessment of the vascular wall and quantitative measurement of key parameters, including neointimal area and burden. These modalities can also characterize different tissue components and are therefore widely used in clinical practice. Nevertheless, intracoronary imaging requires invasive catheterization, which carries a risk of secondary endothelial injury and may further promote neointimal growth. In addition, image acquisition and interpretation remain partly operator‐dependent, introducing potential measurement variability. Compared with intracoronary imaging, non‐invasive coronary imaging modalities such as CCTA avoid catheter‐related vascular complications. However, their limited spatial resolution restricts accurate quantification of neointimal burden after stent implantation. Future diagnostic strategies may benefit from hybrid intracoronary catheters integrating OCT, IVUS, and molecular imaging probes. Such platforms could simultaneously assess vascular morphology and inflammatory cell activity, shifting diagnostic evaluation from purely structural imaging toward combined structural and functional assessment. Further miniaturization of catheter systems may also reduce procedure‐related vascular complications [256, 257]. In non‐invasive imaging, high‐resolution CCTA combined with artificial intelligence‐based reconstruction algorithms may improve diagnostic performance in high‐risk patients and facilitate assessment of therapeutic responses.

DES remain the standard interventional strategy for preventing neointimal hyperplasia. Meanwhile, extensive preclinical research has identified numerous candidate therapeutics and innovative approaches capable of suppressing neointimal growth. Nevertheless, many promising preclinical strategies fail to achieve the expected efficacy or acceptable safety profiles after clinical translation. Several factors contribute to this translational gap. Small‐animal vascular injury models commonly used in drug studies do not fully reproduce the pathological characteristics of human coronary artery disease. In addition, many clinical studies lack direct quantification of neointimal burden by IVUS or OCT and instead rely on indirect surrogate markers. Such endpoints cannot provide direct evidence that candidate therapies attenuate neointimal hyperplasia in patients. Furthermore, the commercialization of novel DES platforms requires rigorous evaluation by regulatory agencies, including the FDA, EMA, and NMPA. Complex approval processes and substantial development costs therefore constitute major barriers to translating promising preclinical findings into clinical practice.

Future research should improve the existing translational framework in several respects. First, large‐animal stent implantation models should be more widely adopted to better recapitulate the pathogenesis of human ISR. Second, therapeutic efficacy should be evaluated using composite endpoints that integrate serological biomarkers with direct intracoronary OCT or IVUS measurements of neointimal hyperplasia. Such a tiered assessment strategy would reduce reliance on indirect serum markers and provide more robust evidence for determining whether candidate therapies directly inhibit neointimal growth in patients. Finally, therapeutic development targeting core pathological mechanisms should move beyond single‐target approaches toward coordinated interventions involving multiple interacting pathways.

## Author Contributions

**Kaixu Wang**: conceptualization, data curation, software, visualization, writing – original draft. **Daiyu Zhang**: writing – original draft. **Yu Hu**: visualization. **Yixin Zhao**: software. **Xinling Wang**: data curation. **Long Jiang**: writing – review and editing. **Meixiu Jiang**: conceptualization, funding acquisition, project administration, supervision, writing – review and editing. All authors have read and approved the final manuscript.

## Funding

This study was supported by the Natural Science Foundation of Jiangxi Province (grant no.: 20252BAC240143), the Health Commission of Jiangxi province Science (grant no.: 202311127), and the Jiangxi Province Key Laboratory of bioengineering drugs (grant no.: 2024SSY07061).

## Ethics Approval

The authors have nothing to report.

## Conflicts of Interest

The authors declare no conflicts of interest.

## Data Availability

The authors have nothing to report.
